# Evaluation of chimeric proteins for serological diagnosis of brucellosis in cattle

**DOI:** 10.14202/vetworld.2021.2187-2196

**Published:** 2021-08-24

**Authors:** Aitbay K. Bulashev, Bakytkali K. Ingirbay, Kanatbek N. Mukantayev, Alfiya S. Syzdykova

**Affiliations:** 1 Department of Microbiology and Biotechnology, Faculty of Veterinary and Livestock Technology, S. Seifullin Kazakh Agrotechnical University, Nur-Sultan, 010011, Kazakhstan; 2 Laboratory of Immunochemistry and Immunobiotechnology, National Center for Biotechnology, Nur-Sultan, 010000, Kazakhstan

**Keywords:** *Brucella*, chimeric protein, enzyme-linked immunosorbent assay, serological potential

## Abstract

**Background and Aim:**

An accurate diagnosis of *Brucella*-infected animals is one of the critical measures in eradication programs. Conventional serological tests based on whole-cell (WC) antigens and detecting antibodies against pathogen-associated lipopolysaccharide might give false-positive results due to the cross-reactivity with other closely related bacteria. This study evaluated the serological potential of *Brucella* spp. chimeric outer membrane proteins (Omps) as antigens in an indirect enzyme-linked immunosorbent assay (i-ELISA).

**Materials and Methods:**

The chimeric gene constructs of the most immunodominant regions of *Brucella* Omps 25+31, 25+19, and 19+31 were cloned into the pET28a expression vectors and transformed into *Escherichia coli* BL21 (DE3). The serological potential of chimeric proteins compared with single recombinant Omps (rOmps)19, 25, and/or 31 were studied on blood serum samples of (i) a rabbit immunized with killed *Brucella* *abortus* 19WC, (ii) mice immunized with single rOmps, (iii) cows seropositive for brucellosis by rose Bengal test, and (iv) cattle naturally and/or experimentally infected with brucellosis.

**Results:**

*E. coli* BL21 actively produced *Brucella* chimeric rOmps, the concentration of which reached a maximum level at 6 h after isopropyl-b-D-1-thiogalactopyranoside stimulation. Target proteins were antigenic and expressed in an active state, as recognized by rabbit anti-*B. abortus* antibodies in an i-ELISA and western blotting. Murine antibodies against the single rOmps reacted with chimeric antigens, and conversely, antichimeric antibodies found their epitopes in single proteins. *Brucella* chimeric rOmps showed higher antigenicity in blood sera of seropositive cattle kept in the hotbed of the infection and/or experimentally challenged with brucellosis than single proteins.

**Conclusion:**

*Brucella* chimeric recombinant outer membrane proteins could be a potential antigen candidate for developing an ELISA test for accurate diagnosis of bovine brucellosis.

## Introduction

Brucellosis is one of the most widespread zoonosis globally that significantly reduces livestock productivity and possesses a danger to human health. It remains endemic in some countries of the Middle East, Africa, South and Central America [1], South [2], and Central Asia, including the Republic of Kazakhstan (RK) [3]. A prompt and accurate diagnosis of infected animals is a critical element in the fight against brucellosis. The rose Bengal test (RBT), agglutination test (AT), and complement fixation test (CFT) are used for diagnosing bovine brucellosis in RK. Commercial enzyme-linked immunosorbent assay (ELISA) kits based on smooth *Brucella* lipopolysaccharide (S-LPS) were also used in the serological diagnosis of bovine brucellosis in the period from 2008 to 2013, when vaccination was canceled and “test and slaughter” was declared as the main method for combating brucellosis. The number of cattle positive for brucellosis by ELISA, a year after its introduction into practice, increased by an average of 7.3 times, but the epidemic situation remained tense [4]. These data suggest that large numbers of healthy cattle have been mistakenly identified as infected due to antibodies against S-LPS of bacteria closely related to *Brucella* spp. [5].

Among the nonpolysaccharide, *Brucella* cell components that can minimize cross-reactions are proteins [6], including outer membrane proteins (Omps). Early studies have shown that native proteins [7] and non-LPS antigens [8] can be used to solve cross-reactivity problems and differentiate infected from vaccinated cattle. Advances in genetic engineering have also eliminated difficulties in antigen preparation and the biohazard risks associated with cell culture and opened up new prospects for the study of *Brucella* recombinant Omps (rOmps) diagnostic value. It was found that the use of a single rOmp31 [9,10], rOmp28 [11], rOmp25, and/or rOmp31 [12] provides the specificity for indirect ELISA (i-ELISA) but significantly reduces its sensitivity. The combined use of *Brucella* rOmps10, 19, and 28 imparted a higher sensitivity to the assay in testing AT-positive blood serum samples [13]. The i-ELISA based on a combination of rOmps25, 28, and 31 could even differentiate mice antibodies to virulent *B. melitensis* from antivaccine and/or nonspecific as well as cross-reactive ones [14]. However, these encouraging results have not been confirmed in productive animals. Previous studies showed that antibodies specific to rOmps19, 25, and 31 could also be found in more than half of the animals maintained in a brucellosis-free herd for as long as 10 months after revaccination with *Brucella abortus* 19 [15]. The use of i-ELISAs that detect *Brucella* anti-rOmps separately reduced the sensitivity of the test. These findings led us to speculate that a combination of the rOmps could be a reliable antigen for developing an ELISA test to identify infected heifers before initial vaccination better. Thus, a chimeric (fusion) antigen comprising the most diagnostically important regions of several proteins and synthesized by a single producer strain would provide high accuracy for the analysis and the relative cheapness of a diagnostic kit.

This study was designed to obtain *Brucella* chimeric rOmps that might be used as an effective antigen for serological diagnosis of bovine brucellosis.

## Materials and Methods

### Ethical approval

All activities involving animals were approved by the Animal Ethics Committee, Faculty of Veterinary and Livestock Technology, S. Seifullin Kazakh Agrotechnical University (KazATU), and performed in accordance with the Guidelines for Accommodation and Care of Animals: Species-specific provisions for laboratory rodents and rabbits (Interstate Standard, GOST 33216-2014).

### Study period and location

The study was conducted from March to December 2020. Sixty outbred male mice (9-10 weeks, 20-25 g body weight) were maintained under appropriate hygienic conditions in the vivarium of KazATU, Nur-Sultan, Kazakhstan.

### Bacterial strains, plasmids, and culture media

The strains *Escherichia* *coli* DH5a and BL21 (DE3) (Novagen, USA) and the plasmids pGEM-TEasy (Promega, Medison, USA) and pET28 (Novagen) were used in this study. The cell cultures were grown on Luria–Bertani (LB) broth and LB agar media (Thermo Fisher Scientific, Waltham, USA) with ampicillin and/or kanamycin (100 and 50 μg/mL, respectively) (Sintez, Kurgan, Russia).

### Design of genetic constructs

On the basis of studies by Tibor *et al*. [16], Wergifosse *et al*. [17], and Vizcaino *et al*. [18], the most immunodominant regions of the *Brucella* spp. Omps19, 25, and 31 were selected, respectively. The search for the amino acid sequences of the selected proteins fragments was conducted using the NCBI PubMed databases (http://www.ncbi.nlm.nih.gov/pubmed). Bioinformatic analysis was conducted using the Vector NTI v.11.5 software package (Invitrogen, USA). Codon optimization of the nucleic acid sequences for the *E. coli* K12 expression system was performed for the efficient expression of recombinant proteins. Based on the nucleotide sequences of the genes, three chimeric genetic constructs were created that are responsible for the synthesis of *Brucella* spp. fusion proteins in the following combinations: Omp19 + 25, Omp19 + 31, and Omp25 + 31.

### Gene synthesis

The chimeric genes of *Brucella* Omps selected fragments were synthesized by Macrogen (Seoul, South Korea) and obtained in lyophilized form with a concentration of 50 ng/μL. Each gene contained restriction sites and six histidine codons (6His-tag) at 5′-end.

### Cloning, expression, and purification of the chimeric recombinant proteins

*E. coli* DH5α was transformed with each of the three genes to generate a preparative amount of DNA and determine their nucleotide sequences. The bacterial cells grown on LB agar were analyzed using a polymerase chain reaction (PCR) using Taq polymerase (Thermo Fisher Scientific) and M13 primers (Promega, Medison, USA). The positive clones were used for DNA purification and sequencing. The BigDye Terminator reagent kit (Thermo Fisher Scientific) was used for sequencing. The resulting genes were cloned into the pET28 plasmid using the EcoRI and XhoI restriction sites.

BL21 (DE3) competent *E. coli* was transformed with the gene inserted plasmid vector pET-28 by electroporation using a MicroPulser (Bio-Rad, USA) under the following conditions: 100 ng of plasmid per 50 mL cell suspension at 2.5 kV, 25 μF, and 200 ohms. The duration of the electroporation was 5.0 ms. The transformed cells were incubated in 950 μL superoptimal broth (Thermo Fisher Scientific, Vilnius, Lithuania) at 37°C for 1 h with shaking at 150 rpm. Then, 50 μL cells were plated on LB agar containing kanamycin as a selective antibiotic and grown at 37°C for 16 h. Single colonies of transformants were cultured in LB broth with kanamycin. In the middle of the logarithmic phase of bacterial growth (absorbance at λ=600 nm, OD600=0.6), 0.1 mM inducer, isopropyl-β-D-1-thiogalactopyranoside (IPTG) (Sigma-Aldrich, St. Louis, USA), was added, and the culture was incubated at room temperature for 16 h with shaking. Bacterial cells were collected by centrifugation at 6000× *g*, 4°C, 7 min.

### Lysis of bacterial cells and chromatographic purification of target proteins

Bacterial cells were lysed using OmniRuptor 4000 Ultrasonic Homogenizer (Omni International, Georgia, USA) at 24 kHz in a pulsed mode (10 pulses per second) in ice-cold buffer (20 mM NaCl, 20 mM HEPES, and 0.1 mM phenylmethylsulfonyl fluoride, pH 7.5). Recombinant proteins were purified by metal chelate chromatography (Ni2+) on a 1 mL HisTrapTM HP column (GE Healthcare, USA). For this, inclusion bodies containing the recombinant proteins were harvested by centrifugation, and the supernatant was removed. The residue was dissolved in buffer (20 mM Tris-HCl, pH 8.0, containing 8 M urea, 500 mM NaCl) and resonated. Insoluble material was pelleted by centrifugation and discarded. The protein solution was loaded onto a nickel–nitrilotriacetic acid column (bed volume 2 mL) and equilibrated with the same buffer. The column was washed with ten volumes of equilibration buffer (20 mM Tris-HCl, pH 8.0, containing 8 M urea; 500 mM NaCl; 20 mM imidazole). A linear imidazole gradient (20-500 mM) was used for the final elution of the recombinant proteins from the chromatographic column. Fast protein liquid chromatography was used for protein purification. Protein fractions were detected at λ = 280 nm. The target proteins were confirmed by western blot using anti-His Tag mouse monoclonal antibody (MAb) conjugated with horseradish peroxidase (HRP, Thermo Fisher Scientific).

#### Sodium dodecyl sulfate–polyacrylamide gel electrophoresis (SDS-PAGE) and western blot

The purified rOmps19+25, 19+31, and/or 25+31 were run on 12% SDS-PAGE gel. After electrophoresis, samples were transferred to nitrocellulose membrane (GE Healthcare Life Sciences, UK) at 2 mA/cm^2^ constant current for 60 min using a semidry electro blot© containing the transfer buffer (25 mM Tris-HCl, 192 mM glycine, and 20% methanol). The membrane was blocked with 1% bovine serum albumin (Sigma-Aldrich, St. Louis, MO, USA) for 1 h at 4°C, washed thrice with 0.05% phosphate-buffered saline (PBS), Tween-20 (PBS-T), and incubated with diluted rabbit anti-*B. abortus* 19WC serum (1:100) for 2 h at 4°C. The membrane was washed with 0.05% PBS-T, incubated with HRP-labeled goat anti-rabbit antibodies (Jackson ImmunoResearch, West Grove, USA) for 1 h at 4°C, and washed with 0.05% PBS-T. The proteins were developed in a 4-chloro-1-napthol solution (Sigma-Aldrich, St. Louis, USA).

*Brucella* rOmp19 [19], rOmp25, and rOmp31 [20] obtained in our previous research were used as single proteins for a comparative study of chimeric proteins’ serological potential.

### Serum samples

A total of 166 cattle sera were tested. Of these, 77 serum samples were from cows seropositive for brucellosis by RBT from a new infection focus (Zhitikara rayon, Kostanay oblast, RK), 34 brucellosis-positive sera were obtained from the National Reference Center for Veterinary Medicine, Ministry of Agriculture, RK, and 12 serum samples were from cattle experimentally infected with the virulent strain *B. abortus* 544; this was kindly provided by Professor K. Tabynov, Head of the Laboratory for the Prevention of Infectious Diseases, Research Institute for Biological Safety Problems, RK. Forty-three sera were obtained from unvaccinated seronegative cattle by RBT, AT, and CFT (Bukhar-Zhyrau rayon, Karaganda oblast, RK) kept in a brucellosis-free farm was used as control samples.

Rabbit anti-*B. abortus* 19 whole-cell (WC) serum obtained in our previous study was used [15]. Mice anti-*Brucella* chimeric Omps sera were obtained as follows. Three groups of outbred mice, with 10 mice per group, were immunized twice subcutaneously with 25 μg rOmp19+25, rOmp19+31, and/or rOmp25+31, respectively. Incomplete Freund’s adjuvant (IFA) (Sigma-Aldrich, Taufkirchen, Germany) on day 0 and PBS (pH 7.2-7.4) on day 14 were mixed in equal amounts with immunogens. Blood was sampled from the tail vein on day 28; sera isolated after centrifugation were used to determine antibody titer against the used immunogens as well as individual (single) proteins (rOmps19, 25, and/or 31) using i-ELISA. As a negative group, ten mice without any injection were regarded.

Three groups of five outbred mice each were used to get mice anti-*Brucella* single rOmps sera. The mice were immunized with 25 μg of the following proteins: rOmp19 (Group I), rOmp25 (Group II), and rOmp31 (Group III) according to the scheme described above. As a negative group, five mice without any injection were used.

### Determination of the antigenicity of the chimeric proteins by i-ELISA using anti-*Brucella* WC sera

The wells in polystyrene plates (Thermo Fisher Scientific) were coated with rOmp19 + 25, rOmp19 + 31, and rOmp25 + 31 at 1.0 μg/mL in bicarbonate buffer, pH 9.6, and incubated at 4°C overnight. Rabbit antiserum to *B. abortus* 19WC and/or serum samples of cattle positive to brucellosis and/or experimentally infected with *B. abortus* 544 were diluted in eight protein-coated wells, starting with 1:100 in PBS-T; the plate was maintained at 37°C for 1 h. Goat anti-rabbit immunoglobulin G (IgG)-peroxidase (Jackson ImmunoResearch, West Grove, USA) and/or rabbit antibovine antibodies (Sigma-Aldrich, St. Louis, USA) were used as secondary antibodies. The dilution of the antiserum was taken to determine the titer of the rabbit antibodies, the optical density (OD) of which was two or more times higher than the OD of the negative control serum at a dilution of 1:100. The cutoff value of i-ELISA for cattle was set at twice the average OD (492 nm) value of *B. abortus*-negative sera (n=43) at a 200-fold dilution [21].

### Determination of the antigenicity of the chimeric proteins via i-ELISA using anti-*Brucella* single rOmps sera

The polystyrene plate wells were coated with chimeric proteins, and mice anti-rOmps19, 25, and 31 sera were diluted as described above. In addition, antibodies bound to the plate were detected with HRP-conjugated rabbit anti-mouse IgG (Sigma-Aldrich, St. Louis, MO, USA). The cutoff value for the assay was calculated as the mean-specific OD plus three standard deviations (SDs) for five sera from nonimmunized mice assayed at a 1:100 dilution.

### Determination of the antigenicity of the single rOmps by i-ELISA using anti-*Brucella* chimeric Omps sera

Briefly, the wells of a polystyrene plate were coated with rOmps19, 25, and/or 31, and mice antisera against chimeric rOmps were diluted in wells coated with homologous single Omps as described above. The cutoff value for the assay was calculated as the mean-specific OD plus three SDs for 10 sera from nonimmunized mice assayed at a 1:100 dilution. All assays were conducted in triplicate and repeated thrice.

### Statistical analysis

Comparisons between the OD mean values were analyzed using Student’s t-test. Statistical analysis of antibody titers was conducted according to a previously described method [22]. Statistical significance was assumed at the p<0.05 level.

## Results

**Figure-1:**
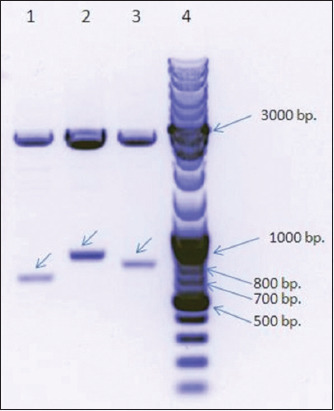
Restriction fragments of *Brucella* chimeric outer membrane proteins genes and pUC57 vector. (1) Omp25+31 (653 bp.); (2) Omp19+25 (806 bp.); (3) Omp19+31 (734 bp.); (4) markers.

**Figure-2:**
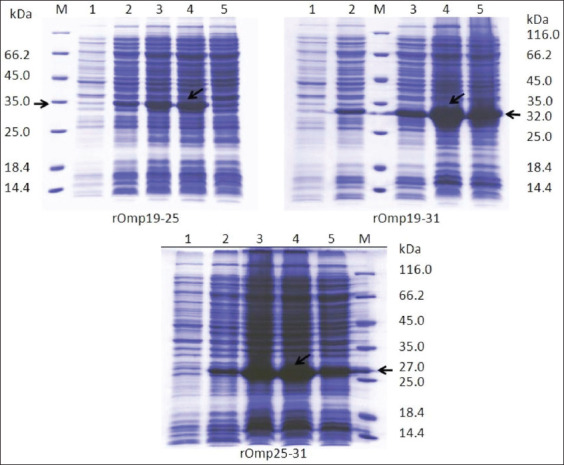
Sulfate–polyacrylamide gel electrophoresis analysis of Brucella chimeric recombinant outer membrane proteins. (1) Before IPTG-induction; (2) 2 h after IPTG-induction; (3) 4 h after IPTG-induction; (4) 6 h after IPTG-induction; (5) after overnight induction; M=Markers.

The amino acid sequences of the selected immunodominant regions of *Brucella* Omps19, 25, and 31 were as follows (Table-1). The restriction of the DNA inserts encoding the following three fusion proteins: Omp19+25, Omp19+31, and Omp25+31, and subsequent electrophoretic analysis revealed DNA bands with the expected sizes of 806, 734, and 653 bp, respectively (Figure-1). The resulting DNA fragments were purified from the gel, ligated to the expression vectors, and transformed into the *E. coli* BL21 (DE3) cells. Bacterial cells were grown on LB broth, and the addition of IPTG induced the expression of the chimeric proteins. SDS-PAGE data of cell lysates are shown in Figure-2. As shown in Figure-2, 6 h of post-induction incubation was optimal to produce target proteins with higher volumetric yield.

**Table-1:** Amino acid sequences of selected *Brucella* Omps.

*Brucella* spp. Omps	Amino acid sequences
Omp31	KAETKVEWFGTVRARLGYTATERLMVYGTGGLAYGKVKSAFNLGDDASALHTWSDKTKAGWTLGAGAEYAINNNWTLKSEYLYTDLGKR
Omp25	WAKKSKDGLEVKQGFEGSLRARVGYDLNPVMPYLTAGIAGSQIKLNNGLDDESKFRVGWTAGAGLEAKLTDNILGRVEYRYTQYGNKNYDLAGTTVRNKLDTQDFRVGIGYKF
Omp19	LAGCQSSRLGNLDNVSPPPPPAPVNAVPAGTVQKGNLDSPTQFPNAPSTDMSAQSGTQVASLPPASAPDLTPGAVAGVWNASLGGQSCKIATPQTKYGQGYRAGPLRCPGELA
	NLASWAVNGKQLVLYDANGGTVASLYS

Omps=Outer membrane proteins

**Figure-3:**
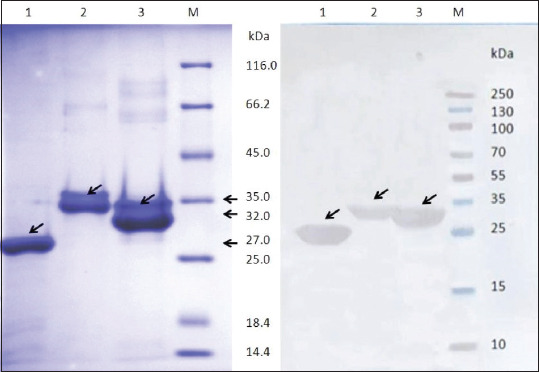
Sulfate–polyacrylamide gel electrophoresis (SDS-PAGE) analysis of chimeric recombinant outer membrane proteins (rOmps) produced by *Escherichia coli* BL21 (a) and antigenicity of the expressed proteins by western blot (b). The proteins were separated by 12% SDS-PAGE and stained with Bromophenol blue R250 (a). The antigenicity of the proteins was elucidated with rabbit anti-*Brucella abortus* 19WC serum with a titer 1: 51 200 by indirect enzyme-linked immunosorbent assay (b). The arrows indicate chimeric rOmps. Lane 1: rOmp25+31; Lane 2: rOmp19+25; Lane 3: rOmp19+31; Lane M=Markers.

An electrophoretogram and western blot of *Brucella* chimeric rOmps purified by metal chelate chromatography (Ni2+) are given in Figure-3. The target proteins have an apparent molecular weight of 35, 32, and 27 kDa for rOmps19 + 25, 19 + 31, and 25 + 31 by electrophoresis, respectively. The specificity of the obtained proteins for *Brucella* spp. was confirmed by the results of immunoblot analysis using hyperimmune serum obtained on day 45 from a rabbit by immunization with phenol-killed *B. abortus*19WC.

The same rabbit antiserum was used to study the comparative antigenicity of *Brucella* chimeric and/or individual proteins (Table-2). Anti-rOmp19+31 and anti-rOmp25+31 antibodies in the blood serum of a rabbit hyperimmunized with inactivated bacterial cells were detected up to a serum dilution of 1:12,800, whereas the antibodies titer against rOmp19+25 and single recombinant proteins (rOmps19 and/or 25) did not exceed 1:1,600. These data indicated that used fusion rOmps were expressed in *E. coli* BL21 in an active form. The expression of chimeric proteins was also confirmed by western blot using His Tag MAb (data not shown).

**Table-2:** Antigenicity of *Brucella* spp. rOmps in rabbit anti-*B. abortus* 19WC serum.

*Brucella* spp. rOmps used as antigens in an indirect enzyme-linked immunosorbent assay					

Individual recombinant proteins			Chimeric recombinant proteins		
	
rOmp19	rOmp25	rOmp31	rOmp19+25	rOmp19+31	rOmp25+31
Titers of anti-*B. abortus* 19WC antibody					
1:1,600	1:1,600	1:6,400	1:1,600	1:12,800	1:12,800

rOmps=Recombinant outer membrane proteins, *B. abortus*=*Brucella abortus*

**Figure-4:**
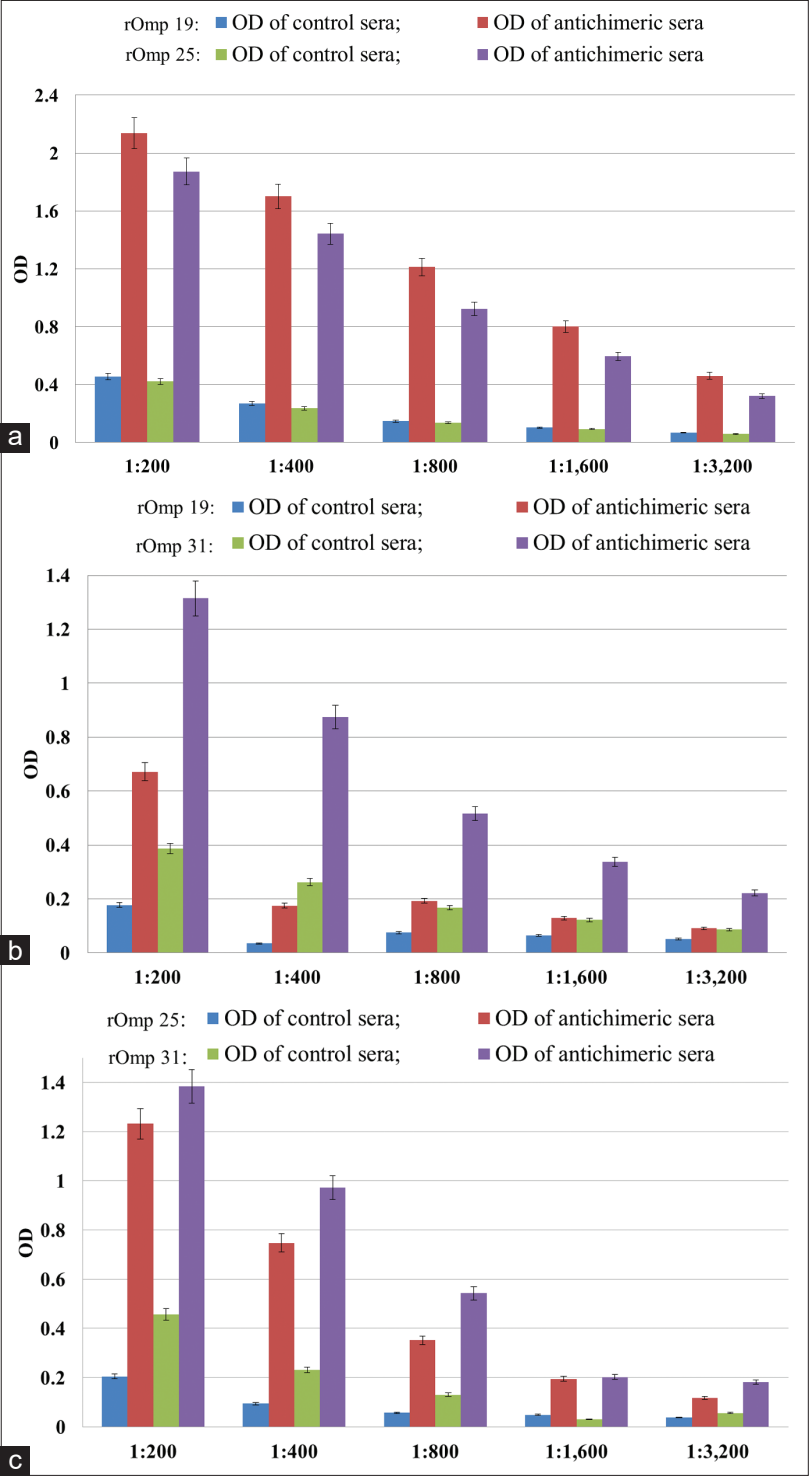
Antigenicity of *Brucella* single recombinant outer membrane proteins (rOmps) in mice anti-chimeric proteins sera yielded day 28. The results of indirect enzyme-linked immunosorbent assay based on rOmps19, 25 and/or 31 are showed in detail. (a) rOmp19 and rOmp25, (b) rOmp19 and rOmp31, and (c) rOmp 25 and rOmp 31.

Chimeric proteins showed sufficient immunogenicity since in the blood sera of mice taken on day 28 after double injections of immunogens suspended in IFA and then in PBS, antibodies against rOmps19+25, 19+31, and/or 25+31 were detected up to titers 1:3,940 (+32.0%; −24.2%), 1:1,840 (+52.6%; −34.4%) and 1:1,040 (+65.9%; −39.7%), respectively. Note that the immunogenicity of rOmp19+25 was significantly higher than that of rOmp19+31 (p<0.05) and rOmp25+31 (p<0.01). These mice antichimeric sera were used to study the antigenicity of *Brucella* single rOmps by i-ELISA (Figure-4).

Antibodies against fusion proteins showed activity against *Brucella* rOmps19, 25, and/or 31, and their titers depend on the type of antiserum and antigen, ranged from 1:170 to 1:800 (+24.0%; −19.7%). Anti-rOmp19+25 antibody was more immunoreactive to homologous single proteins (rOmp19 and rOmp25), than the other two antichimeric antibodies, detecting them up to a serum dilution of 1:800 (+24.0%; −19.7%) and 1: 610 (+15.7%; −13.5%), respectively (p<0.05).

*Brucella* spp. chimeric proteins were recognized by mice antibodies raised against individual rOmps (Table-3). Fragments of the single rOmps used to create fusion proteins retain their antigenicity, as evidenced by antibodies’ binding against the whole single proteins to chimeric antigens. There was no statistically significant difference between the antibody titers of the sera used, and only anti-rOmp31 serum showed higher titers (1:740; p<0.05) to the homologous protein in the structure of rOmp19+31 fusion antigen. The sensitivity, specificity, and accuracy of the rOmps based i-ELISA compared with the RBT are presented in Table-4.

**Table-3:** Antigenicity of *Brucella* chimeric proteins in antisera against the single recombinant outer membrane proteins.

Mice anti-*Brucella* sera used in an indirect enzyme-linked immunosorbent assay					

Antiserum to rOmp19		Antiserum to rOmp25		Antiserum to rOmp31	
rOmp19+25	rOmp19+31	rOmp19+25	rOmp25+31	rOmp19+31	rOmp25+31
1:240	1:240	1:400	1:460	1:740	1:460
(7.2%; –6.7%)	(15.7%; –13.5%)	(15.7%; –3.5%)	(7.2%; –6.7%)	(24.0%; –9.7%)	(24.0%; –9.7%)

**Table-4:** Evaluation of diagnostic values of recombinant outer membrane proteins based i-ELISA compared to RBT.

RBT (+) sera (n=34)	RBT (-) sera (n=43)	Characteristics of i-ELISA				

		Antigens	Sensitivity, %	Specificity, %	Accuracy, %	ODt/ODc
	
i-ELISA (+)	i-ELISA (–)
30	37	rOmp19	88.2	86.0	87.0	4.7±0.4
16	40	rOmp25	47.1	93.0	72.7	2.3±0.1
33	34	rOmp31	97.1	79.1	87.0	3.0±0.3
34	41	rOmp19+25	100	95.3	97.4	4.7±0.1
34	42	rOmp19+31	100	97.7	98.7	5.1±0.1
34	43	rOmp25+31	100	100	100	2.9±0.2

Sensitivity=i-ELISA (+)/RBT (+)×100; Specificity=i-ELISA (–)/RBT (–)×100; Accuracy=i-ELISA (+) plus ELISA (–)/RBT (+) plus RBT (–)×100; ODt=OD of the test samples; ODc=OD of the control samples, i-ELISA=Indirect enzyme-linked immunosorbent assay, RBT=Rose Bengal test

ELISA based on chimeric proteins showed maximum sensitivity with a specificity of 95.3-100% and accuracy of 97.4-100%, whereas these indicators, when using the single rOmps were in the range of 88.2-97.1%, 79.1-93.0%, and 72.7-87.0%, respectively. Judging by the mean ODt/ODc values, rOmp19+31 binds to specific antibodies more strongly than rOmp19+25 (p<0.05) and rOmp25+31 (p<0.01). The serological potential of *Brucella* spp. rOmps was studied on 89 cattle sera, including 77 and 12 *Brucella*-positive samples of cows naturally and experimentally infected with brucellosis, respectively (Table-5).

**Table-5:** Serological potential of *Brucella* spp. rOmps.

*Brucella* spp. rOmps used as antigens in an indirect enzyme-linked immunosorbent assay					

Individual recombinant proteins			Chimeric recombinant proteins		
	
rOmp19	rOmp25	rOmp31	rOmp19+25	rOmp19+31	rOmp25+31
Number of seropositive cows (n=77) from brucellosis-affected farm with anti-rOmps antibodies, heads (%)					
43 (55.8)	35 (45.5)	46 (59.7)	49 (63.6)	73 (94.8)	76 (98.7)
ODt/ODc					
3.0±0.1	3.1±0.1	2.9±0.1	2.7±0.1	4.3±0.2	3.4±0.1
Number of cattle experimentally infected with *Brucella abortus* 544 (n=12) with anti-rOmps antibodies on day 14 post infection, heads (%)					
12 (100)	8 (66.7)	7 (58.3)	12 (100)	12 (100)	12 (100)
ODt/ODc					
6.9±3.0	2.9±1.8	2.5±1.9	2.3±0.4	2.6±0.5	2.6±0.5

rOmps=Recombinant outer membrane proteins

The antigenicity of *Brucella* spp.rOmp19+31 (94.8%) and rOmp25+31 (98.7%) in blood sera of cows kept in the hotbed of a new brucellosis infection was significantly higher compared to rOmp19+25 and individual proteins. Simultaneously, the mean ODt/ODc values in anti-ELISA/rOmp19+31 greatly exceeded those when both single and other chimeric proteins were used in the test (p<0.01). Antibodies against fusion rOmps and/or rOmp19 were detected in serum samples of all experimentally infected animals (on day 14 post-infection); however, ELISA based on rOmp25 and/or rOmp31 showed positive results only in 66.7% and/or 58.3%, respectively.

## Discussion

Despite the importance of brucellosis for veterinary medicine and public health, serological tests developed at the beginning (AT and CFT) and/or in the second half of the last century (RBT, etc.) remain the main diagnostic methods this day. Highly sensitive immunological methods based on the use of labeled antigens and/or antibodies, such as ELISA and lateral flow assay, are now being introduced into the practice of diagnosing brucellosis. The main obstacle to the widespread use of modern tests is the lack of serologically potential antigens specific to *Brucella* spp. Like traditional serological tests, ELISA kits available on the veterinary market are based on the use of pathogen’s S-LPS, which could lead to false-positive results due to antigen cross-reactivity. The search for specific antigens is the key to increasing the diagnostic accuracy of serological tests. Among non-LPS antigens, Omps have been characterized as potential immunoreactive antigens for diagnosis of bovine [12,23,24], ovine, caprine [25], and human brucellosis [26]. *Brucella* spp. Omps, unlike LPS, could (i) differentiate specific antibodies from cross-reactive ones [27], (ii) be produced from harmless producer strains, (iii) have stable antigenic properties, and (iv) improve assay standardization [28]. *Brucella* Omps certainly provide specificity for serological tests but using a single protein reduces ELISA sensitivity [12,29]. This might be related to the fact that all antibodies could not detect a single protein within the overall population. In a study, we found that testing cattle blood sera for antibodies to single proteins (Omp19, Omp25, and/or Omp31) does not identify all seropositive animals. Moreover, antibodies to Omps were detected not only in the infected but also in a particular part of vaccinated animals for a long time after immunization [12,15]. Thus, Omps could only be used to identify infected animals in an unvaccinated herd. Other researchers share our opinion, who argue that the presence of anti-*Brucella* antibodies in unvaccinated animals is always suggestive of infection [30]. Thus, we believe that recombinant antigens comprising several proteins should be used for the reliable detection of infected cattle among non-immune livestock. The main drawback of this approach is the high cost of serological testing, since it will be necessary to have several producer strains and work on the production and purification of two or more recombinant proteins. In addition, each single protein that makes up the combined antigen might have weakly antigenic and/or cross-reacting fragments as well as compete with each other for binding to the ELISA solid phase.

In this study, three types of antigen, each comprising the immunodominant regions of two *Brucella* Omps, were successfully expressed in *E. coli* BL21 (DE3) cells using the pET28 plasmids and their efficacy was assessed in an attempt to increase the sensitivity of the i-ELISA for serological diagnosis of bovine brucellosis. These chimeric proteins, designated rOmp19 + 25, rOmp19 + 31, and rOmp25 + 31 were composed of active serological parts of *Brucella* spp. Omps19, 25, and 31. The recombinant proteins differ from the native ones and may lose some function after expression and/or purification. Besides, western blot procedures might impact their structure. It is impossible not to consider the fact that the immunoreactivity of proteins might be different when comparing *in vitro* to *in vivo* conditions [28]. Thus, the purified chimeric proteins were subjected to relevant studies to verify their validity. *E. coli* BL21 (DE3) actively produced all target proteins, the concentration of which reached a maximum level as early as 6 h after IPTG stimulation of the culture. The i-ELISA and immunoblotting results showed that the tested recombinant antigens are recognized by rabbit anti-*B. abortus* 19WC antibodies, which are evidence of their expression in *E. coli* cells in an active state.

The chimeric antigens were found to be sufficiently immunogenic, since mice immunized with double injections without using complete Freund’s adjuvants showed high levels of antibody titers. An important fact is that antichimeric antibodies also reacted with single proteins, and conversely, murine antibodies to rOmps19, 25, and/or 31 found their epitopes in chimeric rOmps that prove the authenticity of the fusion proteins.

Anti-*Brucella* sera obtained from vaccinated, naturally and/or experimentally infected cattle were used to further verify the diagnostic value of the chimeric antigens by i-ELISA. The results testify that fusion protein contains epitopes against which antibodies are produced in vaccinated and infected animals. The antigenicity of rOmps19+31 and/or 25+31 in postvaccinal and post-infectious cattle sera, as well as in rabbit anti-*B. abortus* 19WC serum, was more pronounced compared to separately taken single Omps and/or rOmp19+25. However, it is interesting to note that the latter fusion antigen was more immunogenic and, additionally, antibodies against it bind better to its constituent proteins. Apparently, the peptides included in rOmps19+31 and 25+31 are immunogenic only in the structure of the cell wall natural proteins. These results suggest the possibility of creating a chimeric protein that might be a good candidate for diagnostic purposes and the development of subunit vaccines. The protective potential of *Brucella* fusion proteins consisting of a trigger factor, Omp31 and Bp26 [31], and four major Omps (Omp16, Omp2b, Omp31, and BP26) [32] has been proven in a mouse model. In comparison with single proteins, the use of chimeric antigens yielded higher sensitivity, specificity, and accuracy for detecting anti-*Brucella* antibodies in cattle serum samples through i-ELISA. The specificity of the assay based on chimeric antigens in testing seropositive animals was slightly lower (by 2%–5%) than using RBT. It should be noted here that WC used in RBT does not exclude false-positive results due to reactions of heterogeneous serum antibodies with *Brucella* S-LPS. Thus, the effectiveness of ELISAs based on chimeric proteins should also be determined when compared with that of the bacteriological analysis, that is, the gold standard for brucellosis diagnosis. Further research will be required to fully assess the diagnostic value of the used immunoassay variants and determine their role in the system of bovine brucellosis control in Kazakhstan.

## Conclusion

The accuracy of animal testing for brucellosis is primarily determined by the specificity of the antigens used in the diagnosis of the disease. Serological tests based on *Brucella* WC and/or LPS could lead to false-positive results due to antigenic similarity of the pathogen to other related bacteria. In this study, we obtained three types of *Brucella* spp. chimeric antigen, comprising the immunodominant regions of two recombinant proteins: rOmp19+25, rOmp19+31, and rOmp25+31. The results showed that chimeric proteins have pronounced immunoreactive properties compared to separately taken single Omps, and might be used to identify infected animals in an unvaccinated herd.

## Authors’ Contributions

AKB: Prepared the manuscript and carried out the sample collection. KNM: Designed the study. BKI: Obtained and purified recombinant proteins. ASS: Carried out laboratory analyses. All authors read and approved the final manuscript.
